# Antibacterial Effect and Mechanism of Chelerythrine on *Xanthomonas oryzae* pv. *oryzae*

**DOI:** 10.3390/microorganisms13040953

**Published:** 2025-04-21

**Authors:** Yi Yan, Jueyu Wang, Na Zhao, Daizong Cui, Min Zhao

**Affiliations:** 1 College of Life Science, Northeast Forestry University, Harbin 150040, China; 2 Key Laboratory for Enzyme and Enzyme-like Material Engineering of Heilongjiang, Harbin 150040, China

**Keywords:** chelerythrine, *Xanthomonas oryzae* pv. *oryzae*, antibacterial action

## Abstract

*Xanthomonas oryzae* pv. *oryzae* (*Xoo*) is a biotrophic bacterial pathogen, which causes devastating bacterial blight disease worldwide. In this study, we thoroughly investigated the antimicrobial effect of the plant-derived extract chelerythrine against *Xanthomonas oryzae* pv. *oryzae* (*Xoo*) and elucidated its mechanism. Chelerythrine is a quaternary ammonium alkaloid with a 2,3,7,8-tetrasubstituted phenanthridine structure, extracted from plants, such as the whole plant of Chelidonium majus, and the roots, stems, and leaves of Macleaya cordata. We found that chelerythrine significantly inhibited the growth of *Xoo* at a concentration of 1.25 μg/mL. Further experiments revealed that chelerythrine interfered with the division and reproduction of the bacterium, leading to its filamentous growth. Additionally, it increased the permeability of *Xoo* cell membranes and effectively decreased the pathogenicity of *Xoo*, including the inhibition of extracellular polysaccharide production, cellulase secretion, and biofilm formation. Chelerythrine induced the accumulation of reactive oxygen species in the bacterium, triggering oxidative stress. The result showed that chelerythrine inhibited the formation of the Z-ring of *Xoo*, interfered with the synthesis of pyrimidine and purine nucleotides, inhibited DNA damage repair, and inhibited the formation of peptidoglycan and lipid-like A, thus interfering with cell membrane permeability, inhibiting carbohydrate metabolism and phosphorylation of sugars, reducing pathogenicity, and ultimately inhibiting bacterial growth and leading to the destruction or lysis of bacterial cells. Altogether, our results suggest that the antimicrobial effect of chelerythrine on *Xoo* exhibits multi-target properties. Additionally, its effective inhibitory concentration is low. These findings provide a crucial theoretical basis and guidance for the development of novel and efficient plant-derived antimicrobial compounds.

## 1. Introduction

Rice is the most important crop worldwide, with more than 163 million hectares planted globally [1,2]. Rice is susceptible to various microorganisms such as bacteria, fungi, and viruses during its growth process [3]. Leaf blight caused by *Xanthomonas oryzae* pv. *oryzae* (*Xoo*) is one of the most lethal rice diseases, which can cause yield losses from 20% to 30% and sometimes as high as 50% [4]. Leaf blight is more prevalent in the southern part of China. In recent years, it has also been found in the northeast regions [5]. Planting resistant rice varieties has been considered the most economical and efficient strategy to control leaf blight. Unfortunately, some of the rice varieties that were once considered resistant varieties, such as those carrying the resistance genes Xa4 and Xa21, have gradually lost their resistance and transformed into pathogenic varieties [6,7]. Hence, fungicide and antimicrobial spraying is still an indispensable and emergency method in controlling rice diseases.

Presently, chemical fungicides are the main method to protect plants from microbial diseases and maintain the quality and safety of crops. Nevertheless, the overuse of chemical fungicides can cause serious environmental problems and health hazards [8,9]. On the other hand, plant-derived antimicrobial extracts are environmentally friendly and do not trigger pest resistance [10]. Hence, the development of new pesticides using plant-derived extracts as raw materials is warranted.

Chelerythrine is a substance belonging to the benzophenanthridine group of alkaloids, which is primarily found in plants such as *Chelidonium majus* and *Macleaya cordata*. Chelerythrine exhibits a wide range of pharmacological activities in the medical field, including anticancer, antibacterial, anti-inflammatory, and antifibrotic effects [11,12,13,14,15]. Chelerythrine also has a potential application in the field of agriculture and has a good inhibitory effect on rice blast [16] and tomato gray mold [17]. Ulrichova and colleagues showed that signal doses of sanguinarine or chelerythrine (i.e., 10 mg/kg/day) can cause acute necrosis of rat liver tissue [18]. It was also found that intravenous injection of a 5 mg/kg dose of chelerythrine can induce ROS and apoptosis in rat cardiomyocytes [19]. The chelerythrine treatment with a dose range of 1.25–10 μM showed significant cytotoxicity and was dose-dependent. Similarly, previous studies have shown that chelerythrine exhibits strong cytotoxicity against various cell lines within a dose range of 2.5–20 μM, including HL-7702 cells (i.e., liver cell lines derived from normal human liver cells, also known as L02 cells), human cancer cell lines (i.e., Caki and 786-O) HeLa, embryonic fibroblasts, H1299 cells, and A549 cells (i.e., human epithelial cell lines derived from lung cancer tissue) [20]. Chelerythrine induces apoptosis via ROS-mediated endoplasmic reticulum stress and STAT3 pathways in human renal cell carcinoma [21]. Presently, the protective role of chelerythrine against rice bacterial diseases is not well studied, especially its antibacterial activity against *Xoo*. Furthermore, the underlying inhibitory mechanism is yet to be elucidated.

We have been working on the mechanism underlying the inhibition of rice diseases by chelerythrine. Our previous study was confused about its ability to control fungal diseases [16,22,23], and we have reported some results. Chelerythrine has moderate antiviral activity against various viruses. Most relevant studies indicate that quercetin can prevent virus replication. Especially for viruses that use protein kinase C during the onset of the disease, quercetin may be a potential inhibitor. So far, there have been few studies on the antiviral effects of chelerythrine. Owing to its broad-spectrum antimicrobial properties, we hypothesize that chelerythrine may have the same inhibitory effect on rice bacterial diseases. Preliminary studies have shown that chelerythrine exhibits good bacterial inhibitory activity against *Xoo*, which provides strong support for further in-depth studies on its mechanism of action.

In this study, we investigated the effects of chelerythrine on *Xoo* in vitro by performing various experiments. We observed the morphological changes in the bacteria, analyzed cell membrane permeability, evaluated reactive oxygen species (ROS) levels, assessed pathogenicity, and analyzed the differential proteins by tandem mass spectrometry-labeled (TMT)-based quantitative proteomics. We aimed to elucidate the pathways underlying the inhibitory effect of chelerythrine on *Xoo*. Our findings provide a theoretical basis for the control and treatment of rice leaf blight due to *Xoo* and for the control of bacterial plant diseases. We believe that the application of chelerythrine in the field of plant disease control will be more promising with the deepening of research.

## 2. Materials and Methods

### 2.1. Bacterial Strains, Culture Conditions, and Chemical Agents

*Xoo* strain GX13 was provided by Prof. G. Q. Yuan (Guangxi University, Guangxi, China) [24].

Chelerythrine was extracted from *Chelidonium majus* in our laboratory and identified as chelerythrine using liquid chromatography [23]. HPLC-grade formic acid, methanol, and acetonitrile were obtained from Thermo Fisher Scientific (Waltham, MA, USA). All other chemicals and reagents were of analytical grade.

### 2.2. In Vitro Test for Antibacterial Activity of Chelerythrine Against Xoo

Ping Yang’s method was used to determine the antimicrobial activity [24]. The minimum inhibitory concentration (MIC) and minimum bactericidal concentration (MBC) of chelerythrine against *Xoo* were determined using broth dilution methods. The final concentrations of chelerythrine solution were 0.63, 1.25, 2.5, 5, 10, 15, 20, 25, 30, 35, 40, 60, and 80 µg/mL. The solutions were mixed with a 10^7^ colony-forming unit (CFU)/mL suspension of *Xoo* and cultured for 24 h, and its growth was observed. A 100 µL liquid aliquot from each treatment was plated on NA to determine the MIC. The nutrient broth was centrifuged, and the bacteria were grown on an NA plate for 48 h. To determine the MBC, the nutrient broth was centrifuged and grown on an NA plate within 96 h.

Growth determination assays were performed on NB with final chelerythrine concentrations of 0.5×MIC and 1×MIC, then mixed with 10^7^ CFU/mL concentrations of *Xoo*. Distilled water was used instead of chelerythrine in the control group. All inoculation broths were cultured for 76 h. The OD_600_ was detected every 4 or 8 h until bacterial growth reached the stationary stage. Each treatment was performed in triplicate.

### 2.3. Effect of Chelerythrine on the Cell Structure of Xoo

#### Scanning Electron Microscopy (SEM)

The cell structure was observed using the method of Kang Shimo, with some improvements [25]. Exponential-phase *Xoo* cells were treated with chelerythrine at final concentrations of 1×MIC and incubated at 28 °C for 24 h and 48 h. Then, the cells were washed with PBS (pH 7.4), fixed with 2.5% glutaraldehyde, and dehydrated with ascending concentrations of ethanol. The cells were then freeze-dried with tert-butanol and coated with gold. The ultrastructure was observed by SEM using a Hitachi S-4800 field emission microscope (Tokyo, Japan).

### 2.4. Effect of Chelerythrine on Membrane Permeability of Xoo

To better observe the experimental phenomena, the bacterial concentration was 10^8^ CFU/mL. In this experiment, all the subgroups were the chelerythrine group (0.5×MIC, 1×MIC, and 2×MIC) and the control group (no chelerythrine addition).

Cultured to logarithmic growth phase, centrifuged at 8000 rpm for 10 min and resuspended with sterilized medium and grouped according to the requirements, 500 µL of 2-nitrophenyl-β-D-galactopyranose (ONPG) at a concentration of 0.03 mol/L was added to each treatment, mixed well, and incubated for 4 h at 28 °C, 130 rpm with shaking. Each treatment was repeated thrice. Each treatment was centrifuged at 4000 rpm for 10 min, and the absorbance was measured at 420 nm from the supernatant. The changes in the generated o-nitrophenol (ONP) were found.

### 2.5. Effect of Chelerythrine on the Pathogenicity of Xoo

#### 2.5.1. Determination of Extracellular Polysaccharide (EPS) Production

The effect of cypermethrin on EPS production was evaluated using the phenol–sulfuric acid method. The bacterial suspension was diluted to the optical density at 600 nm of 0.1 and centrifuged at 4000 rpm for 10 min after shaking at 28 °C for 24 h. Of note, 5 mL of the supernatant was mixed with 2 mL of Sevag’s reagent (chloroform/n-butanol, 3:1), and the proteins were precipitated by vigorous shaking, incubated for 1 min, and centrifuged. The precipitate was discarded, and 1 mL of supernatant was mixed with 3 mL of 95% ethanol. This was shaken well and allowed to stand for 20 h at 4 °C. The mixture was then centrifuged at 4000 rpm for 10 min. The precipitate contained the crude polysaccharide, which was dried and dissolved in 5 mL of distilled water, 1 mL of 5% phenol, and 5 mL of concentrated sulfuric acid. After the reaction was completed, the absorption was detected at 490 nm. Each treatment and control group was performed in triplicate.

#### 2.5.2. Extracellular Cellulase Activity Assays

Each culture dish was inoculated with 1 µL of *Xoo*. The suspension was placed in the center of the plate and incubated at 28 °C for 72 h. Using the ratio of hydrolytic zone diameter (D) to colony diameter (d), the extracellular cellulase activity under different concentrations of chelerythrine was calculated. Each treatment was performed in triplicate.

#### 2.5.3. Effect of Chelerythrine on Biofilm Formation

The mixture from each treatment group and control group was added to a 24-well plate, with 3 replicates from each group, and incubated in a 28 °C constant temperature incubator for 48 h. This allowed the formation of biofilms on the plate wall. The bacterial solution was poured into a 24-well plate and gently rinsed with sterilized saline to remove excess bacterial body. This was air-dried, and 2 mL of 0.1% crystalline violet staining was added for 30 min. The plate was washed with saline twice to remove excess. Lastly, 2 mL of 30% glacial acetic acid was added to dissolve crystal violet, and the plates were incubated at room temperature for 10 min to determine the absorbance at 570 nm. The crystal violet should be completely dissolved; otherwise, the results may be inaccurate.

### 2.6. Cell Mobility Test

The bacterial suspension in the logarithmic growth period was centrifuged and resuspended. Of note, 1 µL of bacterial suspension was added into the middle of an NA plate, which contained 0.3% *w*/*w* agar. After the bacterial solution was absorbed, the plates were incubated at 28 °C for 72 h. The diameter of the bacterial swimming circle was measured, and the average value was calculated. Each treatment was performed in triplicate.

### 2.7. Effect of Chelerythrine on Pyruvate Content of Xoo

Of note, 2 mL of bacterial solution was taken from each treatment and centrifuged at 4000 rpm for 10 min. The supernatant was taken to determine the pyruvic acid in the culture solution as per the manufacturer’s protocol. The change in pyruvic acid content in the culture solution of each treatment was determined. Each treatment was performed in triplicate.

### 2.8. Determination of ROS

Each treatment and control group was incubated under shaking conditions at 130 rpm and 28 °C for 24 h. Then, it was centrifuged at 4000 rpm and 4 °C for 10 min. The supernatant was removed, and cells were obtained. Cells were washed thrice with sterile 0.01 M PBS (pH 7.4) and resuspended with the same PBS to obtain an optical density at 600 nm of 0.2. Cells were incubated with DCFH-DA at 10 mM for 30 min at 37 °C in the dark. Cells were resuspended with 1 mL of PBS (pH 7.4). ROS was evaluated using TECAN, a multifunctional enzyme labeler with excitation/emission settings of 488/525. Each treatment was performed in triplicate.

### 2.9. TMT Quantitative Proteomic Sample Preparation

Bacteria in the logarithmic phase were obtained by centrifugation. The fresh culture was resuspended, and permethrin was added to obtain a final MIC of 0.5. This was incubated under shaking conditions at 28 °C and 130 rpm for 8 h. Cells were obtained by centrifugation, washed thrice with sterile 0.01 M PBS (pH 7.4), and stored in a −80 °C refrigerator for some time after rapid freezing in liquid nitrogen. Three biological replicates were performed for each sample using the cypermethrin-free group as a control group.

### 2.10. Protein Extraction, Quantification, Enzymolysis, and TMT Labeling

Samples were thawed and transferred to an MP vibrating tube. An appropriate amount of extraction buffer (1% SDS, 200 mM DTT, 50 mM Tris HCl, pH 8.8 containing protease inhibitor) buffer was added and vortexed. The high-throughput tissue grinder was used to vibrate 3 times for 40 s each time. Samples were kept on ice for 30 min and vortexed every 5 min for 5–10 s. The samples were incubated at 100 °C for 10 min and cooled on ice. The samples were centrifuged at 12,000× *g* and 4 °C for 20 min, and the supernatant was taken. Precooled acetone was added in a ratio of 1:4, and the solution was allowed to precipitate overnight at −20 °C. Then, the solution was centrifuged at 12,000× *g* and 4 °C for 20 min. The next day, the supernatant was removed, and 90% precooled acetone was added for precipitation. The mixture was mixed well and centrifuged, and the supernatant was removed. This process was repeated twice. The precipitate was dissolved with protein lysate (8 M urea + 1% SDS, containing protease inhibitor). This was centrifuged at 4 °C and 12,000× *g* for 20 min to obtain the supernatant containing the protein solution.

Protein content was determined using the Bradford method. Proteins were diluted 5-fold with 100 triethylammonium bicarbonate (TEAB) and digested with trypsin (Promega, Madison, WI, USA) at an enzyme–substrate ratio of 1:50 (*w*/*w*).

The TMT reagent was removed at −20 °C, restored at room temperature, and centrifuged. Then, acetonitrile was added, the solution was centrifuged, and a tube of TMT reagent was added for every 100 µg of polypeptide, and incubated at room temperature for 2 h. Hydroxylamine was added and the solution was kept at room temperature for 30 min. An equivalent amount of labeled product was added to each group in a tube and pumped out using a vacuum concentrator.

### 2.11. Instrumental Analysis

The samples prepared were separated using a Thermo Scientific Vanquish Flex Binary UHPLC system (Waltham, MA, USA). Firstly, UPLC loading buffer solution (2% acetonitrile and ammonia water adjusted to pH 10) was added to redissolve polypeptide samples. Then, the reversed-phase C18 chromatographic column (Acquity uplc beh C18 Column 1.7 µm, 2.1 mm × 150 mm, Waters, Milford, MA, USA) was used for high pH liquid phase separation. Mobile phase A consisted of 2% acetonitrile (pH adjusted to 10 using ammonia), whereas mobile phase B consisted of 80% acetonitrile (pH adjusted to 10 using ammonia). The flow rate was 200 μL/min, the detection wavelength was 214 nm, and the gradient was 48 min. Based on the peak type and time, the distillate was concentrated by vacuum centrifugation, dissolved in mass spectrometry loading buffer (2% acetonitrile and 0.1% formic acid), and analyzed in the second dimension.

### 2.12. Data Analysis

The raw file of MS offline was submitted to the Proteome Discoverer server, and the established database was selected and searched. The identified proteins were screened and evaluated for differences. The *p*-value and difference multiple (FC) of the t-test were selected as the selection. Additionally, the proteins with *p* < 0.05, FC > 1.2, or FC < 0.83 were selected as the upregulated and downregulated proteins with statistical significance and differences in abundance.

### 2.13. Quantitative Real-Time PCR

The total RNA was extracted from the control and chelerythrine-treated *Xoo* using the total RNA extraction kit (Tiangen Biotech, Beijing, China). cDNA synthesis was performed using the ReverTra Ace qPCR RT Kit (Toyobo, Japan). Roche LightCycler480 real-time PCR system (Roche, Indianapolis, Indiana, USA) was used for gene expression analysis using primeScript™RT reagent kit with gDNA Eraser (Perfect Real Time) (Takara, Japan), primer (Table S1), and 1 ng cDNA template under the following settings: initial denaturation at 95 °C for 30 s, 45 cycles at 95 °C for 5 s, and 30 s at 60 °C; then, the melting curve was 5 s at 95 °C, 60 s at 60 °C, and finally at 95 °C for 30 s. The housekeeping gene gyrB was used to normalize the data as an internal control. Relative gene expression analysis was performed in triplicate.

### 2.14. Bioinformatics Analysis

The Gene Ontology (GO) database http://www.geneontology.org (accessed on 1 September 2021) was used to classify and group the identified proteins. The differential expression protein (DEP) functions were analyzed based on functional information provided by the KEGG database http://www.genome.jp/kegg/ (accessed on 1 September 2021).

### 2.15. Statistical Analysis

All experiments were performed in triplicate. The results are expressed as mean ± standard deviation. Statistical analyses were performed by analysis of variance and Duncan test using SPSS 26.0 software (SPSS Inc., Chicago, IL, USA). In all cases, a *p*-value of <0.05 was considered statistically significant. Asterisks are used to indicate statistically significant differences between groups (*p* ≤ 0.05; *p* ≤ 0.01).

## 3. Results

### 3.1. In Vitro Test for Antibacterial Activity of Chelerythrine Against Xoo

When the concentration of chelerythrine was higher than 1.25 μg/mL, the culture medium was clear at 10^7^ CFU/mL of *Xoo* bacterial solution (initial concentration), suggesting that bacterial growth was inhibited. Plates were coated with 100 μL of the clarified medium and incubated at 28 °C, and the growth of the bacteria was observed after 48 h and 96 h, respectively. The MIC and MBC of chelerythrine against *Xoo* were 1.25 μg/mL and 40 μg/mL, respectively.

To further determine the inhibitory effect of chelerythrine on *Xoo*, the growth curves of *Xoo* under different concentrations of chelerythrine treatment were plotted.

**Figure 1:**
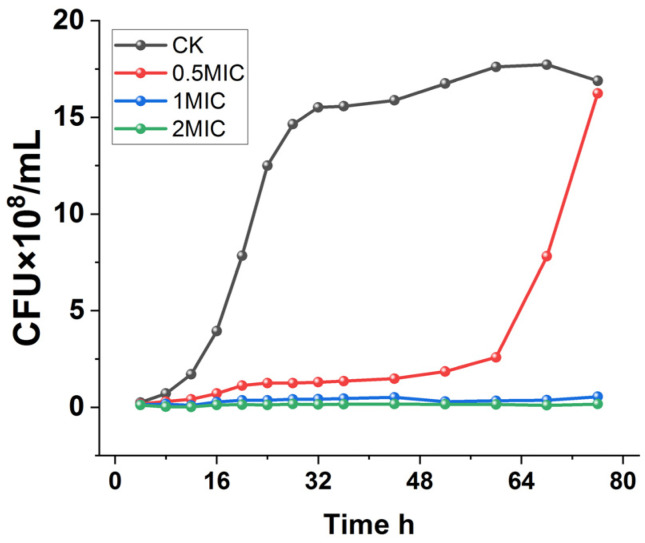
Growth curves of *Xanthomonas oryzae* pv. *oryzae* after treatment with different concentrations of chelerythrine.

As shown in Figure 1, *Xoo* in the control group entered the logarithmic phase after 12 h of incubation and grew slowly after 28 h. On the other hand, the 0.5×MIC treatment group entered the logarithmic phase after 60 h, and the logarithmic phase was delayed by 48 h compared with that of the control group. Furthermore, the culture medium was always present in a clarified state in the 1×MIC and 2×MIC treatment groups, and the growth of the bacteria was completely inhibited.

### 3.2. Effect of Chelerythrine on the Cell Structure of Xoo

**Figure 2:**
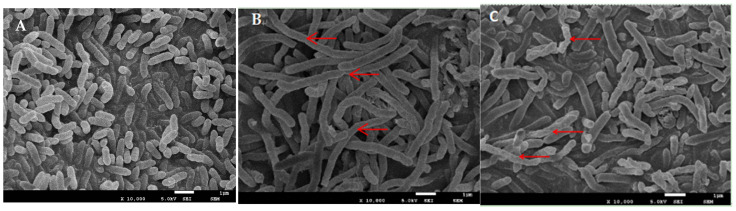
SEM image of *Xoo* ((**A**): SEM image of *Xoo* untreated with chelerythrine; (**B**): SEM image of *Xoo* treated with 1×MIC chelerythrine for 24 h; (**C**): SEM image of *Xoo* treated with 1×MIC chelerythrine for 48 h).

Scanning electron microscopy of the nontreated bacilli revealed that they were rod-shaped with straight, rounded ends, intact, and clear, and had relatively smooth surfaces (Figure 2A). The bacteria treated with the 1×MIC concentration for 24 h were obviously elongated with filamentous growth (Figure 2B). Additionally, a few of them showed rough and wrinkled surfaces, with an extension of time up to 48 h. The degree of wrinkles on the surface of the bacteria increased, and some of them appeared to be broken (Figure 2C).

We hypothesized that chelerythrine can affect the division and reproduction of *Xoo*. Hence, the bacteria grew in a filamentous form, which ultimately led to their breakage and death.

### 3.3. Effect of Chelerythrine on the Membrane Permeability of Xoo

**Figure 3:**
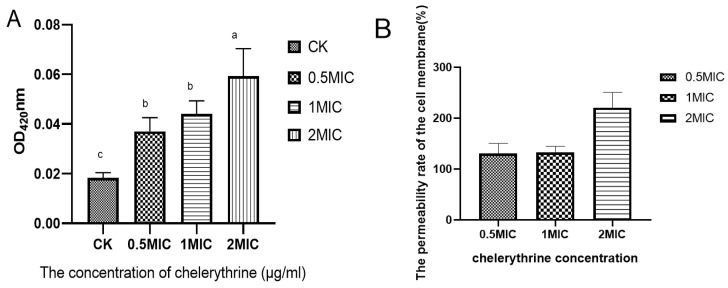
(**A**): The effect of chelerythrine on the permeability of the inner membrane of *Xoo*. (**B**): The ratio of the cell membrane permeability rate of each treatment group to that of the control group. a, b, c,: Different letters indicate statistical differences at the level of *p* < 0.05 between different treatment groups and the control group.

Chelerythrine treatment of *Xoo* for 4 h resulted in the production of o-nitrophenol, which increased as the concentration increased. The results are shown in Figure 3A. The differences between each treatment group and the control group reached a level of significance. We hypothesized that chelerythrine significantly increases the permeability of the cell membrane of *Xoo* in a short time. Then, the permeability improvement rate of each treatment group compared with the control group was calculated. The results are shown in Figure 3B. The cell membrane permeability rate of each treatment group increased by more than 100%, and that of the high-concentration treatment group was even higher than 200%.

### 3.4. Effect of Chelerythrine on Extracellular Cellulase of Xoo

Extracellular cellulase enables pathogenic bacteria to invade the host and spread further within its body. In cellulase experiments (Table 1), the ratio of hydrolyzed circles (D) to colony diameter (d) reached significant differences in the chelerythrine treatment and control values of 1.667, 1.531, 1.446, and 1.879, indicating that chelerythrine can inhibit the ability of *Xoo* to produce extracellular cellulase. Additionally, the higher the concentration, the better the inhibitory effect.

### 3.5. Effect of Chelerythrine on the EPS Content of Xoo

After 24 h of chelerythrine treatment, the EPS production of *Xoo* decreased as the treatment concentration increased, which reached a significant level of difference between the treatment groups and the control group (*p* < 0.01) (Table 2). This suggests that chelerythrine can inhibit the production of EPS of *Xoo*, thus reducing its pathogenicity.

### 3.6. Effect of Chelerythrine on Biofilm Formation of Xoo

**Figure 4:**
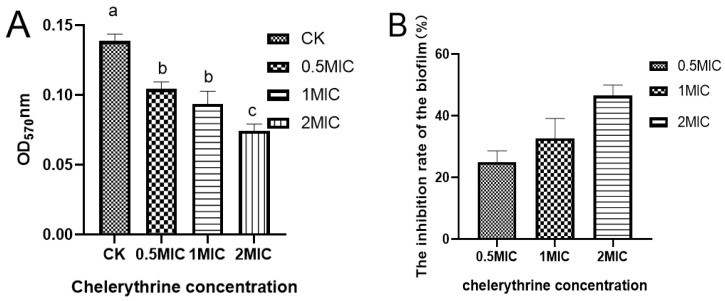
(**A**): The effect of chelerythrine on the biofilm formation of *Xoo*. (**B**): The inhibition rate of cell membranes in each treatment group compared with the control group. a, b, c: Different letters indicate statistical differences at the level of *p* < 0.05 between different treatment groups and the control group.

After 48 h of static incubation, the difference between the absorbance of the treated group and the control group was significant, suggesting that chelerythrine can inhibit biofilm formation (Figure 4A). The difference between the 0.5×MIC and 1×MIC treatment groups was not significant. However, the absorbance value of the 1×MIC treatment group was smaller than that of the 0.5×MIC. Additionally, the difference between the 2×MIC treatment group and other groups was extremely significant, suggesting that chelerythrine can inhibit biofilm formation at a lower concentration. Additionally, the higher the concentration, the better the inhibitory effect. The inhibition rate of the biofilm was shown in Figure 4B.

### 3.7. Effect of Chelerythrine on Pyruvate Content of Xoo

**Figure 5:**
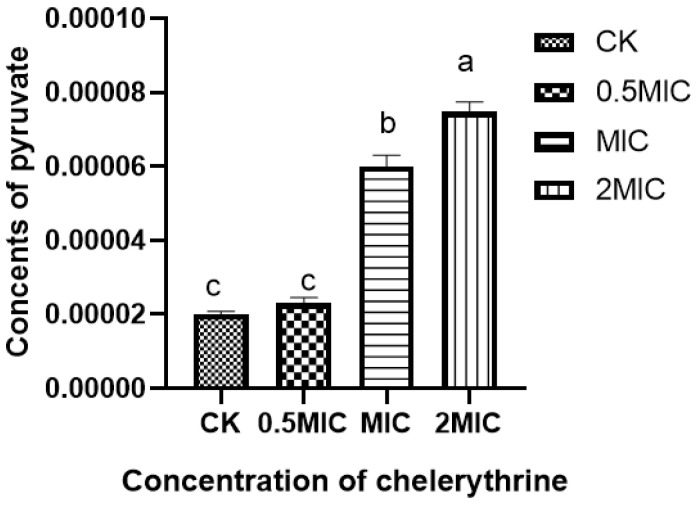
The effect of chelerythrine on the pyruvate content of *Xoo*. a, b, c: Different letters indicate statistical differences at the level of *p* < 0.05 between different treatment groups and the control group.

After chelerythrine treatment, the results are shown in Figure 5, the pyruvate content in the bacteria of the 0.5×MIC treatment group increased slightly compared with the treatment group but did not reach a significant level of difference. Additionally, both the 1×MIC and 2×MIC treatment groups reached a significant level of difference compared with the control group. This indicates that chelerythrine can lead to the abnormal accumulation of pyruvate in the body and affect carbohydrate metabolism.

### 3.8. Effect of Chelerythrine on Cell Mobility of Xoo

**Figure 6:**
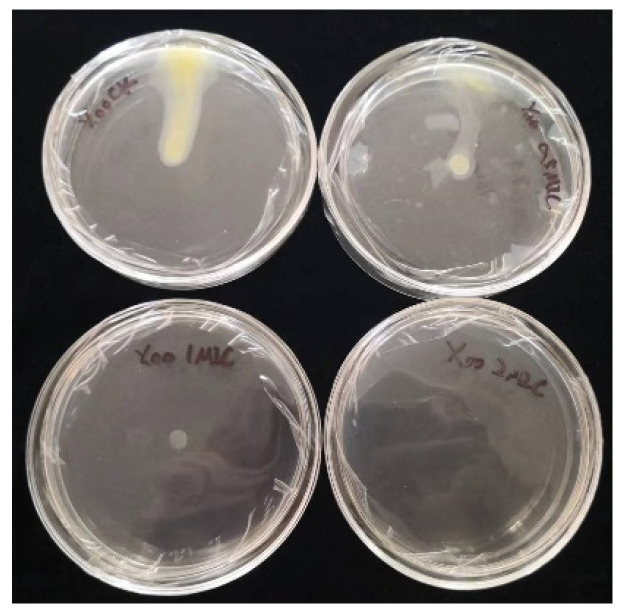
The effect of chelerythrine on the cell mobility of *Xoo*.

Inoculation of bacteria on chelerythrine plates at different concentrations showed that the growth of the bacteria was significantly inhibited (Figure 6). Additionally, the higher the concentration of the inhibitor, the stronger the inhibition effect. At a 0.5×MIC concentration, the growth and motility of the bacteria were attenuated. At a 1×MIC concentration, the growth of the bacteria was significantly decreased, and the motility was almost lost. At a 2×MIC concentration, the growth of the bacterium was almost stagnant(Table 3). Thus, we found that chelerythrine can inhibit the growth of the bacterium and affect its motility.

### 3.9. Effect of Chelerythrine on ROS of Xoo

**Figure 7:**
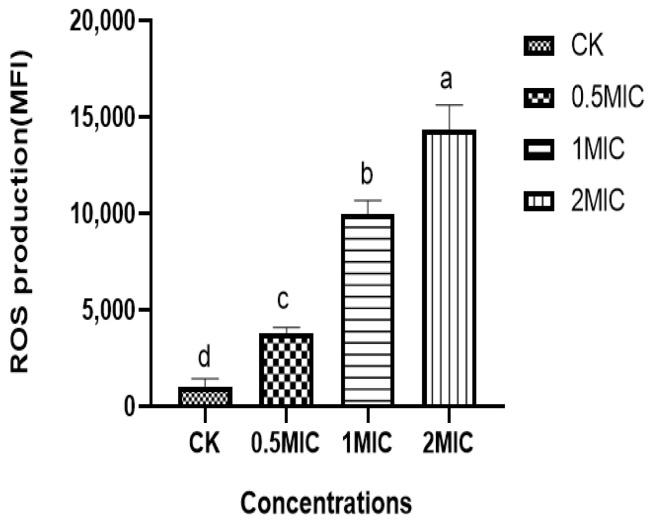
The effect of chelerythrine on the ROS of *Xoo*. a, b, c, d: Different letters in Figure 7 indicate statistical differences at the level of *p* < 0.05 between different treatment groups and the control group.

We found that the ROS content in the bacteria of the chelerythrine-treated group was significantly increased, and the difference between the treatment groups and the control group was extremely significant (*p* < 0.01) (Figure 7), suggesting that chelerythrine disrupts the oxidative balance in vivo and leads to the excessive accumulation of ROS in the *Xoo* cells, leading to oxidative stress.

### 3.10. Proteomic Analysis

#### 3.10.1. Protein Identification and Quantification

In this experiment, data were used for the statistical analysis of differential proteins to determine the potential antimicrobial effect of chelerythrine on *Xoo*. The truncation values of expression variants (multiple changes ≥ 1.2, ≤0.83, *p*-value < 0.05) were used for differential protein analysis. In total, 1147 differential proteins were selected, of which 225 were upregulated, whereas 922 were downregulated (Supplementary Table S2).

#### 3.10.2. GO Enrichment Analysis of DEPs

According to the GO enrichment analysis, the highest level of enrichment was transmembrane signaling receptor activity, followed by exoribonuclease activity, protein methyltransferase activity, and exoribonuclease activity.

#### 3.10.3. DEPs Involved in Cell Membrane and Cell Division

The expression of cell division-related proteins, including FtsA, FtsQ, FtsX, FtsE, ZapA, and MreB, was downregulated. The expression of proteins related to cell membrane formation, including LpxC, LpxL, LpxK, and LpxH, was downregulated. Similarly, the expression of peptidoglycan-forming ligases, including MurC, MurD, MurE, MurF, and MurG, was downregulated.

#### 3.10.4. DEPs Involved in Oxidative Stress and DNA Repair

The expression of proteins involved in cellular oxidative homeostasis, including AhpC and KatE, was upregulated. Additionally, the expression of AhpF, Ohr, Dps, and TrxA was downregulated. The expression of proteins associated with recombinant DNA repair, such as RuvB, UuvA, UvrC, UvrD, RecG, UNG, and UDG, was downregulated.

#### 3.10.5. DEPs Involved in Chemotaxis and Bacterial Movement

DEPs associated with chemotaxis, such as MCP, CheA, CheB, CheW, and CheD, were upregulated after the action of chelerythrine. Additionally, the flagellum, as a motor organ, exhibited abnormal expression with the upregulated expression of FlgI, FliO, and FliD, and downregulated expression of FliI, FliJ, FliN, FlgG, FlgF, and FlgL after treatment with chelerythrine.

DEPs involved in the expression of glucokinase, phosphofructokinase, and fructose-1,6-bisphosphatase in the glycolysis pathway were downregulated. Aconitase in the citric acid cycle was upregulated, isocitrate dehydrogenase and fenvalerate lyase were downregulated, and gluconolactam 6-phosphate endolipase and ribulose 5-phosphate isomerase in the pentose phosphate pathway were downregulated. Additionally, in the fructose PTS system, the expression of EIIB and EIIC was downregulated. The expression of NADH dehydrogenase subunits NuoB, NuoC, NuoF, and NuoI was downregulated. Additionally, the expression of cytochrome C oxidase subunits CyoC and CyoA was downregulated, and the expression of cytochrome C reductase subunit Cyt1 was downregulated.

#### 3.10.6. DEPs in Nucleotide Synthesis

The expression of PyrD, PyrE, and PyrF in the pyrimidine nucleotide de novo synthesis pathway was downregulated. The proteins PurE, PurT, and PurH in the purine nucleotide synthesis pathway were significantly downregulated.

Additionally, biotin, similar to many key enzymes in living organisms, is involved in several material metabolic pathways in organisms. In the synthesis pathway of biotin, the expression of BioC, BioF, and BioB was significantly downregulated.

## 4. Discussion

### 4.1. Effects of Chelerythrine on Cell Division and Cell Membrane of Xoo

Cell division is a complex biological process regulated by several proteins. It includes the accurate identification of the division site, localization of the Z-ring, and the regulation and contraction of the inner membrane and cell wall. Abnormal or missing expression of some essential proteins during division can lead to the development of slender filamentous morphology and, ultimately, cell death.

In this study, *Xoo* exhibited significant elongation after exposure to chelerythrine. This was consistent with the elongation observed in *Xoo* following berberine treatment and the effect of resveratrol on *E. coli* [24,26].

The division and proliferation of bacteria are guided by the FtsZ protein, which plays a crucial role in bacterial division. FtsZ, in conjunction with various proteins, such as FtsA, FtsQ, and ZapA, facilitates cytoplasmic division and cell separation [27,28], which is a significant target for developing novel bacteriostatic agents. In this study, the expression of cleavage proteins such as FtsA, FtsQ, and ZapA was downregulated. This downregulation affects the dynamic assembly of FtsZ and inhibits the formation of the Z-ring, preventing normal bacterial division. As a result, the bacteria elongate into narrow filamentous structures, ultimately leading to cell death. Rai et al. [29] reported that curcumin induced filamentation in Bacillus subtilis 168, indicating that it inhibited bacterial cytoplasmic division. This inhibition ultimately led to the arrest of cell division, which is consistent with the results of the present study.

The bacterial cell membrane components of Gram-negative bacteria include lipid-like A and peptidoglycan. The abnormal expression of key proteins involved in lipid-like A synthesis, such as LpxC, LpxL, LpxK, and LpxH, affects cell membrane biosynthesis and growth, thus affecting its function [30,31,32,33]. Additionally, these proteins can be targeted by drugs to inhibit bacterial growth. Variations in the structure, regulation, and synthesis of peptidoglycan affect cell integrity and morphology. In the present study, we found that the downregulation of key proteins involved in peptidoglycan synthesis, such as MurC, MurD, MurE, MurF, and MurG, affects the morphology of the bacterium and the function of the cell membrane. Pyrazolopyrimidine analogs are highly effective against *E. coli* and Pseudomonas aeruginosa MurC enzymes, selectively inhibiting peptidoglycan biosynthesis. Genetic studies have confirmed that pyrazolopyrimidines target MurC. Additionally, the inhibition of MurC enzymes leads to bacterial cell death [34], which is consistent with the findings of the present study.

### 4.2. Effect of Chelerythrine on Oxidative Stress and DNA Repair of Xoo

Chelerythrine-treated cells showed a considerable increase in ROS levels, which were positively correlated with their concentration. Antioxidant defense systems, including thioredoxin and peroxidase, in microorganisms scavenge ROS and repair oxidative damage [35]. The results indicated that chelerythrine induced the overexpression of AhpC, KatE, and Ohr to reduce ROS and inhibited the expression of AhpF and TrxA to exert bacteriostatic effects. In *E. coli*, AhP and KatE are the main enzymes that scavenge hydrogen peroxide [36]. Additionally, the formation of a complex between AhpC and AhpF increases cellular salvage [37]. Ohr proteins are crucial components of the antioxidant system [38]. Trx exhibits an anti-oxidative effect via the scavenging of ROS and interacts with peroxide oxidoreductase [39]. Chelerythrine can lead to intracellular ROS accumulation, induce *Xoo* oxidative stress, and induce AhpC, KatE, and Ohr to protect bacterial cells from ROS. Concurrently, it can also inhibit the activities of AhpF and TrxA, which cause disorders in the antioxidant enzyme system, leading to an imbalance in redox stress and an increase in ROS that exceeds the capacity of oxidative enzymes to scavenge them, thus leading to cellular damage.

Additionally, excessive ROS can damage DNA. DNA repair is important to maintain normal cellular physiological functions. RuvB and RuvA proteins are highly conserved and involved in DNA recombination and DNA double-strand break repair. UvrC is a crucial component of the nucleic acid repair enzyme UvrABC-type proteins, which performs damage removal and DNA resynthesis [40]. UvrD plays a role in DNA mismatch repair and nucleotide excision repair [41]. The recG deconjugating enzyme can direct DNA synthesis during DNA double-strand break repair [42]. Lastly, DNA glycosylase is one of the most important enzymes in base excision repair. It can be used to complete DNA mismatch repair by removing uracil residues from the DNA and then combining them with other proteases [43]. The proteomics results indicated that the expression of proteins related to DNA repair and recombination was downregulated, suggesting that chelerythrine inhibited the DNA repair pathway. This disrupted the integrity and stability of the genetic information of the bacterium, ultimately causing cell damage or death.

### 4.3. Effect of Chelerythrine on the Pathogenicity of Xoo

The pathogenicity of bacteria is closely associated with their extracellular polysaccharides, biofilm formation, extracellular enzyme secretion, and motility. Extracellular polysaccharides facilitate bacterial adhesion to surfaces, aggregation among bacteria, and biofilm structure maintenance. In this experiment, chelerythrine could inhibit the production of extracellular polysaccharides, biofilms, and extracellular cellulases. The proteomics assay revealed a downregulation of the exopolysaccharide xanthan gum biosynthesis glycosyltransferase, GumM, with a downregulation factor of 0.85, which did not reach 0.83 but was significant. Reduced secretion of extracellular polysaccharides inhibits biofilm formation, consequently affecting the pathogenicity of the bacteria. Sridhar Dharmapuri’s findings corroborate these results, showing that GumM is essential for *Xoo* pathogenicity. Its deletion affects bacterial swimming, biofilm synthesis, and EPS production and decreases bacterial pathogenicity [44]. These findings are highly consistent with the findings of the present study experiment. The Type 1 secretion system (T1SS) is prevalent in the protein secretion system of Gram-negative bacteria and is involved in the secretion of drug resistance and pathogenicity factors [45]. Some studies have shown that efflux protein TolC plays an important role in biofilm formation in Escherichia coli [46], and the downregulation of TolC expression observed in the present study also similarly affected biofilm formation.

Bacterial chemotaxis is the motile response to a concentration gradient of a chemical elicitor or repellent. This process involves cell surface chemoreceptors (MCPs) that detect chemotactic ligands, generating signals transmitted to flagellar motility through a series of chemotactic proteins. In the present study, the expression of chemotactic receptor proteins, MCP, CheA, CheB, CheW, and CheD, was upregulated in *Xoo*. This indicates that the upregulated expression of chemotactic proteins in response to leucocyanidin stimulation may generate chemotactic signals, triggering flagellum-driven chemotaxis. However, the expression of most flagellum-related proteins was downregulated after leucocyanidin treatment, resulting in reduced motility. This is consistent with the findings of Huai-Zhi Luo et al. [47], where *Xoo* treated with resveratrol exhibited inhibited flagellar growth, affecting chemotaxis and motility and reducing pathogenicity.

### 4.4. Effect of Chelerythrine on Carbohydrate Metabolism of Xoo

Carbohydrate metabolism is vital for microbial survival. In the present study, the expression of several key proteins involved in carbohydrate metabolism, including glycolysis and the tricarboxylic acid cycle, was downregulated. Chelerythrine-treated *Xoo* exhibited slower growth and lower metabolic activity, potentially extending its survival time.

However, the phosphotransferase system (PTS) in bacteria serves not only to transport and phosphorylate carbohydrates but also to regulate carbon metabolism, which serves catabolism [48]. The PTS comprises cytoplasmic phosphotransferases (enzyme I), histidine phosphate carrier protein (HPr), and a quintuple-dependent variable number of glycan-specific enzyme II complexes (EIIA, EIIB, and EIIC). EIIA and EIIB transfer phosphates from HPr to sugar, whereas EIIC is an endosomal protein responsible for binding and transferring sugar [49]. After chelerythrine treatment, the expression of phosphoenolpyruvate phosphotransferase PtsI and HPr was downregulated, along with EIIB and EIIC, suggesting that chelerythrine could inhibit phosphate-to-sugar conversion, while preventing *Xoo* from using the PTS system pathway to phosphorylate sugar for energy. *Xoo* also possesses a nitrogen phosphotransferase system, downregulated by the expression of PtsN nitrogen regulatory protein, indicating that chelerythrine can inhibit nitrogen metabolism in vivo. These findings suggest that chelerythrine inhibits carbohydrate metabolism and the production of phosphorylated sugars, reducing carbohydrate transport capabilities. Consequently, *Xoo*’s energy supply diminishes, forcing the bacterium into a lower metabolic state, and slowing growth to extend its survival time.

### 4.5. Effect of Chelerythrine on the Nucleotide Metabolism of Xoo

Abnormal nucleotide metabolism can directly affect the viability of the bacterium and is considered a target for bacterial inhibition. In this experiment, three differential proteins associated with pyrimidine nucleotide synthesis were evaluated, including dihydroorotate dehydrogenase PyrD, which is the rate-limiting key enzyme in the pathway of pyrimidine nucleotide synthesis from scratch, orotate phosphoribosyltransferase PyrE, and orotidine 5′-phosphate decarboxylase, which react via several enzyme linkage reactions to ultimately produce uridine monophosphate (UMP), which is a precursor of all pyrimidine nucleotides [50]. The downregulated expression of these enzymes after chelerythrine treatment indicates that chelerythrine can act as an inhibitor, affecting the formation of pyrimidine nucleotides from scratch. Marta Alberti et al. [51] screened and characterized the first selective inhibitor of Mycobacterium tuberculosis divergens dihydroorotic acid dehydrogenase and reported that the inhibitor could suppress its activity. Abdurahman A. Niazy et al. [52] investigated that the pyrE mutant of Pseudomonas aeruginosa PA01 exhibited decreased motility and biofilm-forming ability owing to the disruption of pyrimidine de novo synthesis, which reduces the pathogenicity of Pseudomonas aeruginosa. Knockdown of the pyrF gene results in the loss of the autonomous synthesis of uracil within the bacteria.

Additionally, three differential proteins associated with hypoxanthine nucleotide synthesis were identified in this experiment. Hypoxanthine nucleotide synthesis directly affects the synthesis of other purine nucleotides. Downregulation of N5 carboxyaminoimidazole ribonucleotide mutase PurE, glycosaminoglycan ribonucleotide transcarboxylase PurT, and AICAR transcarboxylase PurH affects purine ribonucleotide synthesis. Munekazu Tagawa et al. [53] reported that purE deficiency leads to protein excretion due to the accumulation of metabolic intermediates that cause protein excretion, possibly due to defects in the purE enzyme. The insertion of a transposon in rice leaf blight bacterium purH leads to its growth defects, probably due to the inability to use sufficient purE [54].

When the bacterium was affected by chelerythrine, the synthesis pathways of pyrimidine and purine nucleotides directly affected DNA and RNA synthesis, cell wall synthesis, glucose metabolism, lipid metabolism, and DNA damage repair, ultimately affecting bacterial survival. These downregulated proteins in nucleotide synthesis can serve as targets for bacterial inhibition or drug therapy, which can be further validated through mutant expression in subsequent experiments.

### 4.6. Effect of Chelerythrine on the Energy Metabolism of Xoo

The pyruvate dehydrogenase complex, found widely in microorganisms, catalyzes the irreversible oxidative decarboxylation of pyruvate to generate acetyl coenzyme (acetyl-CoA) while reducing NAD to NADH. This process links the aerobic oxidation of sugars to the tricarboxylic acid cycle and oxidative phosphorylation, making it a crucial target for energy metabolism [55]. Defects in the pyruvate dehydrogenase complex can cause impaired metabolism and the massive accumulation of pyruvate in microorganisms. Moxley W Chris et al. [56] achieved the accumulation of pyruvate production by knocking out the gene to construct an *E. coli* strain with a pyruvate dehydrogenase complex mutation, indicating that abnormal synthesis of this complex can directly affect pyruvate levels and microbial viability. In this experiment, the expression of pyruvate dehydrogenase complex dihydrolipoamide dehydrogenase (E3) was downregulated by a factor of 0.84, and the difference did not reach the set ratio of 0.83. However, the trend of its change was decreasing. Additionally, due to the decrease in the content of dihydrolipoamide dehydrogenase, the pyruvate content of the bacterium increased. In this experiment, the pyruvate content within the bacteria was also determined after 24 h of chelerythrine treatments. The results showed that the difference between the pyruvate content within the CHE-treated organisms and the control group was extremely significant, which was dose-dependent. This is consistent with the proteomics results.

Oxidative phosphorylation is the primary metabolic pathway for obtaining the energy needed for cell growth and reproduction. The five major protein complexes constituting the electron transport chain drive oxidative phosphorylation [57]. In this experiment, chelerythrine treatment downregulated the expression of cytochrome C oxidase subunits CoxB and CoxC, key components of the electron transport chain, affecting electron transport and ATP synthesis. Some researchers found that treating Vibrio alginolyticus with CU^2+^, Pb^2+^, and low pH downregulated the expression of genes associated with the oxidative phosphorylation pathway, impairing the bacterium’s adhesion ability and cytochrome C oxidase activity, consequently affecting the pathogenicity of the bacteria [58]. Similarly, in this experiment, chelerythrine treatment attenuated the biofilm formation and adhesion abilities of *Xoo*, consistent with the effects observed in Vibrio alginolyticus after treatment with CU^2+^, other treatments, or RNAi.

### 4.7. Effect of Chelerythrine on Biotin Synthesis of Xoo

Biotin is a cofactor involved in various metabolic pathways in the body. Bacteria can synthesize biotin. The final step of biotin synthesis is the insertion of BioB into the biotin sulfur fraction of biotin. Additionally, BioF can concentrate alanine into 8-amino-7-oxononanoic acid (KAPA) and methylate the free carboxylate group malonyl-ACP to form malonyl-ACP methyl ester, which then proceeds to other steps of the biotin synthesis pathway. After chelerythrine treatment, BioB, BioC, and BioF expressions were downregulated, and biotin synthesis was decreased, which affected fatty acids and tricarboxylic acid cycle metabolism, ultimately affecting the survival of bacteria.

### 4.8. Validation of TMT Proteomics Results by RT-PCR

**Figure 8:**
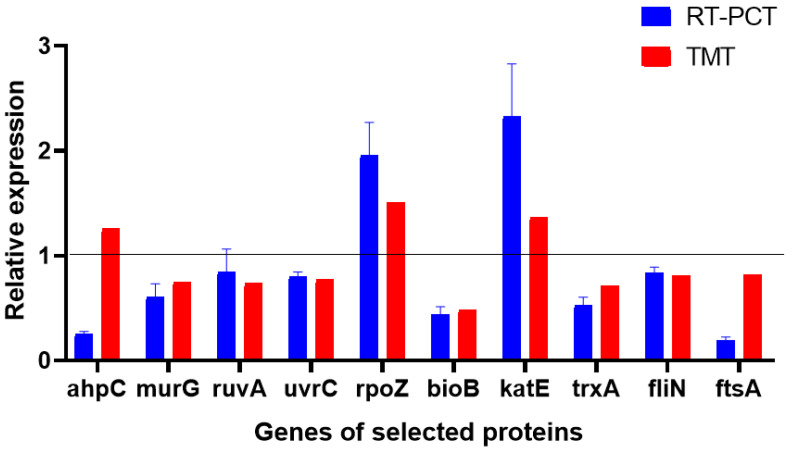
Relative expression levels of transcripts.

The results of the TMT proteomics showed that combined with the DEPs between the control group and the treatment group involved in multiple functions, 10 proteins were selected as target proteins and genes (Figure 8). These proteins were involved in cell wall biosynthesis, translation, transcription, DNA damage repair, cell division, and cell motility. The target proteins and RT-PCR results were analyzed to evaluate the levels of DEPs and mRNAs and confirm the authenticity and accuracy of TMT quantitative proteomics results. We found that nine genes of the target proteins were consistent with the trend of the corresponding protein levels, and one gene was inconsistent with the expression of its corresponding protein. This may be attributed to the post-transcriptional regulation of gene expression. Hence, the results obtained by TMT quantitative proteomics and RT-PCR were consistent, suggesting the reliability of the TMT quantitative proteomics results.

## 5. Conclusions

To summarize, by analyzing the expression of target proteins and genes based on TMT quantitative proteomics analysis, ultrastructural observation, and qRT-PCR, we hypothesized that the antimicrobial mechanism underlying chelerythrine against *Xoo* includes the following: affecting normal cell division by inhibiting Z-ring formation; affecting the formation of lipid-like A and peptidoglycan, thereby affecting cell membrane permeability; inhibiting endogenous antioxidant activity, leading to continuous ROS accumulation, which triggers oxidative stress, DNA damage, altered cell membrane permeability, and other injuries; interfering with the synthesis of purine and pyrimidine nucleotides; blocking DNA repair and recombination pathways, destabilizing the bacterial genome; inhibiting carbohydrates and phosphorylated sugars; and inhibiting virulence, biofilm formation, and motility, thereby decreasing pathogenicity. This comprehensive inhibition ultimately destroys or kills the bacterial cells. To the best of our knowledge, this is the first report to systematically elucidate the mode of action of chelerythrine on *Xoo* at the proteomic level. Because the action of chelerythrine on *Xoo* is multi-targeted, our approach may have potential applications in controlling rice leaf blight disease.

## Figures and Tables

**Table 1:** Effect of chelerythrine on extracellular cellulase of *Xoo*.

Groups	CK	0.5×MIC	1×MIC	2×MIC
D/d	1.879 ± 0.030 a	1.667 ± 0.031 b	1.531 ± 0.008 c	1.446 ± 0.009 d

a, b, c, d: Different letters in Table 1 indicate statistical differences at the level of *p* < 0.05 between different treatment groups and the control group.

**Table 2:** Effect of chelerythrine on the EPS content of *Xoo*.

NameTreatments	CK	0.5×MIC	1×MIC	2×MIC
Extracellular polysaccharide production (µg/mL)	14.122 ± 0.046 a	12.942 ± 0.241 b	12.410 ± 0.160 b	10.905 ± 0.284 c

a, b, c: Different letters in Table 2 indicate statistical differences at the level of *p* < 0.05 between different treatment groups and the control group. Values are represented as mean ± SEM.

**Table 3:** The diameter of bacterial motility.

Groups	CK	0.5×MIC	1×MIC	2×MIC
Diameter of motility (mm)	44.5 ± 0.404 a	44.3 ± 0.448 a	4.63 ± 0.067 b	4.57 ± 0.167 b

a, b: Different letters in Table 3 indicate statistical differences at the level of *p* < 0.05 between different treatment groups and the control group.

## Data Availability

The original contributions presented in this study are included in the article/Supplementary Materials. Further inquiries can be directed to the corresponding authors.

## Supplementary Materials

The following supporting information can be downloaded at: https://www.mdpi.com/article/10.3390/microorganisms13040953/s1, Table S1: primer sequences; Table S2: Selected proteomics results.
