# Supplementary material for: Antibacterial Effect and Mechanism of Chelerythrine on Xanthomonas oryzae pv. oryzae
Source: Microorganisms. 2025 Apr 21;13(4):953. doi: 10.3390/microorganisms13040953 (PMC12029680; doi:10.3390/microorganisms13040953)
Supplement: Supplementary file 1 [file microorganisms-13-00953-s001.zip › microorganisms-3516432-supplementary.pdf]

Antibacterial effect and mechanism of chelerythrine on *Xanthomonas oryzae* pv. *oryzae*

Yan Yi[1],Wang Jueyu[1],Zhao Na[1],Wei Qinghui [2]Cui Daizong[1#],Zhao Min[1#]
[1]Northeast Forestry University, Harbin 150040, China
[2]Institute of Plant Protection, Heilongjiang Academy of Agricultural Sciences, Harbin 150086, China

Table S1 Primer sequences

Gene-ID	NAME	Sequence(5’→3’)	Description
XOC_RS18320 ID:77338777	ahpC-F<br>ahpC-R	TTATCAACACTCAGGTCCAGC<br>ATCAGCACGGACCACTTG	alkyl hydroperoxide reductase subunit
XOC_RS00880ID:77335587	bioB-F<br>bioB-R	TGTTGTCGTCCGCCATGA<br>GACAGCAGTGTGGAGACCT	biotin synthase BioB
XOC_RS18875 ID:77338879	ftsA-F<br>ftsA-R	TGGACGATTCGCAGGAAGG<br>GCACGCACTTGGTGATGTT	Cell division protein FtsA
XOC_RS11660 ID:77337554	fLiN-F<br>fLiN-R	TCGGTGGTGGAACTGGAAC<br>CACATCGGTCAGGCGAATAC	flagellar motor switch protein FliN
XOC_RS18865 ID:77338877	lpxC-F<br>lpxC-R	GCAACGCACGCTCAAGAA<br>ATCGGTTCCAGGTCCACAC	UDP-3-O-acyl-N-acetylglucosamine deacetylase
XOC_RS16860 ID:77338503	katE-F<br>KatE-R	CCCGACGCTGCTGGAAGAT<br>TTGGCAGTGGTGTATTGGCTC	catalase
XOC_RS18895 ID:77338883	murG-F<br>murG-R	GGGATTCGGGATTGGTAAAGAG<br>GAGCGGGATTGGAGATTGG	undecaprenyldiphospho-muramoylpentapeptide beta-N-acetylglucosaminyltransferase
XOC_RS05090 ID:77336352	rpoZ-F<br>rpoZ-R	GCCCGCATTACCGTAGAAG<br>CGGCATTTTCGATCAGCG	DNA-directed RNA polymerase subunit omega
XOC_RS06485 ID:77336611	ruvA-F<br>ruvA-R	GATCCTGGCCTACAAGCAG<br>AGGAAGCCATACAACGACAC	Holliday junction branch migration protein RuvA
XOC_RS20045 ID:77339095	trxA-F<br>trxA-R	GACTTCGATACAGCGGTACTG<br>ATTGACCTTGGCGACCTTC	thioredoxin TrxA
XOC_RS10580 ID:77337353	uvrC-F<br>uvrC-R	GCGTTATTTCGGTCCTTACAC<br>CCACTACAACGTCCAATCTGA	excinuclease ABC subunit UvrC
XOC_RS00025 ID:77335427	gyrB-F<br>gyrB-R	TTACGACTCCAGCAAGATCAC<br>AGCCATCGACCAGGATTTC	DNA topoisomerase (ATP-hydrolyzing) subunit B

Table S2 Details for each differentially expressed protein

Accession	Description	FC (Treated/CK)	P-value (Treated/CK)	KO Name	Regulate
B2SQQ1	DNA-directed RNA polymerase subunit beta	1.663	0.000337	rpoB	up
B2SQQ2	DNA-directed RNA polymerase subunit beta	1.714	0.000128	rpoC	up
A0A0K0GP06	DNA-directed RNA polymerase subunit omega	1.509	0.000635	rpoZ	up
B2SQT4	DNA-directed RNA polymerase subunit alpha	1.448	0.007753	rpoA	up
A0A0K0GPA4	Recombination protein RecR	0.575	0.00028	recR	down
B2STK2	Holliday junction ATP-dependent DNA helicase RuvA	0.737	0.000162	ruvA	down
B2STK0	Holliday junction ATP-dependent DNA helicase RuvB	0.777	0.00002	ruvB	down
A0A0K0GFW3	DNA helicase	0.748	0.00007	recB;uvrD, pcrA;rep	down
A0A0J9WXC9	Replication-associated recombination protein A	0.666	0.000379	dnaX;ycaJ;ruvB	down
B2SJF5	Uracil-DNA glycosylase	0.826	0.005563	UNG, UDG	down
B2SKI8	UvrABC system protein C	0.770	0.000035	uvrC	down
A0A0K0GPE1	ATP-dependent	0.661	0.000069	recG	down

	DNA helicase RecG				
A0A0K0GI60	Alkyl hydroperoxide reductase C	1.257	0.007	ahpC	up
A0A0K0GN92	Catalase	1.375	0.03757	katE	up
A0A0K0GG41	Organic hydroperoxide resistance protein	1.228	0.03407	ohr	up
A0A0K0GMA5	DNA-binding related protein	0.757	0.002168	dps	down
A0A0K0GJX2	Superoxide dismutase	1.295	0.007063	SOD2	up
A0A0K0GGB9	Thioredoxin	0.715	0.0003385	trxA	down
A0A0K0GHQ6	Cell division protein FtsA	0.822	0.000086	ftsA	down
A0A0K0GG44	Cell division ATP-binding protein FtsE	0.760	0.000257	ftsE	down
A0A0K0GGG7	Cell division protein FtsX	0.707	0.000126	ftsX	down
A0A0K0GH96	Cell shape-determining protein MreB	0.722	0.000070	mreB	down
A0A0K0GP47	Cell division protein ZapA	0.597	0.000266	ZapA	down
A0A0K0GHN2	Cell division protein FtsQ	0.666	0.003621	FtsQ	down
A0A0K0GL01	Methyl-accepting chemotaxis protein	2.440678	0.000149	mcp	up
A0A0K0GJR5	Chemotaxis protein	2.225422	3.90E-05	mcp	up
A0A0K0GJ93	Chemotaxis protein	1.977099	9.40E-05	mcp	up
A0A0K0GJK0	Histidine kinase	1.746602	8.00E-06	cheA	up
A0A0K0GJ90	Chemotaxis protein	1.716797	1.10E-05	mcp	up
A0A0K0GJJ2	Chemotaxis protein	1.602161	4.00E-06	mcp	up
A0A0J9WWW	Chemotaxis	1.576049	6.00E-06	mcp;pilJ	up

5	protein				
A0A0J9WX33	Histidine kinase	1.410029	5.00E-06	cheA	up
A0A0K0GIP2	Chemotaxis protein	1.361434	0.001114	mcp	up
A0A0K0GJI1	Probable chemoreceptor glutamine deamidase CheD	1.387735	0.01325	cheD	up
A0A0K0GJG4	Chemotaxis protein	1.279468	0.02063	mcp	up
A0A0K0GJQ4	Chemotaxis protein methyltransferase	1.264078	0.006931	cheR;cheBR	up
A0A0K0GIL7	OmpA family protein	1.286139	0.008359	pal;TC.OOP;motB	up
A0A0K0GJT0	MotA protein	1.287139	0.00012	motA	up
A0A0J9WWX5	Chemotaxis protein	1.212616	0.000359	cheW;cheV;cheY	up
A0A0J9WX32	Protein phosphatase CheZ	1.211475	0.006891	cheZ	up
A0A0J9WXJ3	Flagellar motor switch protein FliN	0.811379	0.001386	fliN	down
A0A0K0GQF9	Methyltransferase	0.755952	0.001684	cheR;cheBR	down
A0A0K0GGA9	Response regulator protein	0.654205	7.00E-06	cheY	down
A0A0J9WXG1	Flagellar P-ring protein	1.883888	0.001161	flgI	up
A0A0K0GGS1	RNA polymerase sigma factor RpoD	1.857302	8.00E-06	rpoD	up
A0A0J9WXJ4	Flagellar protein	1.423831	0.04799	fliO, fliZ	up
A0A0K0GJT0	MotA protein	1.287139	0.00012	motA	up
A0A0J9WWY8	Flagellar hook-associated protein 2	1.227368	0.006689	fliD	up

A0A0J9WXJ3	Flagellar motor switch protein FliN	0.811379	0.001386	fliN	down
A0A0K0GGC4	RNA polymerase sigma factor RpoH	0.821613	0.00113	rpoH	down
A0A0J9WWX8	Basal-body rod modification protein FlgD	0.59505	0.000365	flgD	down
A0A0J9WWX9	Flagellar hook protein FlgE	0.578485	9.90E-05	flgE;flgG	down
A0A0J9WXH1	RNA polymerase sigma-54 factor	0.480593	0.000738	rpoN	down
A0A0J9WWY6	Flagellar hook-associated protein 3	0.530422	0.000137	flgL;fliC, hag	down
A0A0J9WX11	Flagellar FliJ protein	0.414258	0.000501	fliJ	down
A0A0J9WWY0	Flagellar basal body protein	0.456785	0.000926	flgF;flgE;flgG	down
B2SKI3	Tetraacyldisaccharide 4'-kinase	0.728557	0.000716	lpxK	down
B2SMK4	UDP-2,3-diacylglucosamine hydrolase	0.628165	4.40E-05	lpxH	down
A0A0K0GPQ4	Lipid A biosynthesis lauroyl acyltransferase	0.610907	7.00E-06	lpxL, htrB	down
B2SNZ9	UDP-3-O-acyl-N-acetylglucosamine deacetylase	0.604976	6.20E-05	lpxC	down
A0A0K0GPP2	UDP-N-acetylmuramate--L-alanyl-gamma-D-glutamyl-meso-2,6-diaminoheptandioate ligase	0.754848	0.000483	murF;mpl;murD2;murC	down
B2SNZ4	UDP-N-acetylmuramate--L-alanine ligase	0.774107	8.60E-05	murF;mpl;murC	down

A0A0K0GHI6	UDP-N-acetylglucosamine--N-acetylmuramyl-(pentapeptide) pyrophosphoryl-undecaprenol N-acetylglucosamine transferase	0.750532	0.000352	murG	down
A0A0K0GNE4	UDP-N-acetylmuramoyl-L-alanine--L-glutamate ligase	0.615754	0.000393	murE;murF;mpl;murD2	down
A0A0K0GP49	6-phosphofructokinase	0.797	0.001845	pfk	down
A0A0K0GKN7	Glucokinase	0.762	0.000012	glk	down
B2SUX7	Fructose-1,6-bisphosphatase class 1	0.642	0.000025	FBP	down
B2SVN3	Phosphoglucosamine mutase	0.638	0.000450	glmM	down
A0A0K0GGX8	Isocitrate dehydrogenase [NADP]	0.829	0.000352	IDH1, IDH2	down
A0A0K0GNW9	Isocitrate dehydrogenase [NAD] subunit alpha, (Isocitric dehydrogenase) (Nad(+)-specific icdh)	0.787	0.000542	IDH3	down
A0A0K0GRF3	Acetyl-coenzyme A synthetase	1.377	0.001988	acs	up
B2SLM6	Ribose-5-phosphate isomerase A	0.798	0.003685	rpiA	down
A0A0K0GQQ1	Phosphomannomutase	0.789	0.002015	pmm-pgm	down
B2SUX7	Fructose-1,6-bisphosphatase	0.642	0.000025	FBP	down

	class 1				
B2SVN3	Phosphoglucosamine mutase	0.638	0.000450	glmM	down
B2SLM6	Ribose-5-phosphate isomerase A	0.798228	0.003685	rpiA	down
A0A0K0GK86	6-phosphogluconolactonase	0.7889	0.005627	PGLS, pgl, devB	down
A0A0K0GM91	NADH-quinone oxidoreductase subunit F	0.82833	0.000116	nuoF	down
B2SVL7	NADH-quinone oxidoreductase subunit C	0.780278	0.000545	nuoC	down
B2SVL8	NADH-quinone oxidoreductase subunit B	0.813513	0.003819	nuoB	down
A0A0K0GMR5	NADH-quinone oxidoreductase subunit I	0.720419	0.007913	nuoI	down
A0A0K0GLE0	Uridylate kinase	0.820609	0.000322	pyrH	down
B2SHJ1	Orotidine 5'-phosphate decarboxylase	0.801957	0.01846	pyrF	down
A0A0K0GQ76	Orotate phosphoribosyltransferase	0.656918	0.001381	pyrE	down
B2SLB9	Dihydroorotate dehydrogenase (quinone)	0.659676	6.50E-05	pyrD	down
A0A0K0GFM5	Malonyl-[acyl-carrier protein] O-methyltransferase	0.527113	1.10E-05	bioC	down
B2SS67	Biotin synthase	0.485789	1.20E-05	bioB	down
B2SS66	8-amino-7-oxononanoate synthase	0.676639	0.000212	bioF	down
A0A0K0GMK0	N5-carboxyaminoimidazole	0.779769	0.016626	PurE	dwon

	ribonucleotide mutase				
A0A0K0GMN 3	N5-carboxyaminoimidazole ribonucleotide synthase	0.730844	0.000897	purT	down
B2SRB2	Bifunctional purine biosynthesis protein PurH	0.793712	0.000832	PurH	Down
